# An Aquatic Treadmill Alters Lower Limb Walking Dynamics in Typically Developing Children and Children with Cerebral Palsy

**DOI:** 10.3390/s25103220

**Published:** 2025-05-20

**Authors:** Oluwaseye Odanye, Joseph Harrington, Aaron Likens, David Kingston, Brian Knarr

**Affiliations:** Department of Biomechanics, University of Nebraska Omaha, 6001 Dodge St., Omaha, NE 68182, USA; oodanye@unomaha.edu (O.O.); josephharrington@unomaha.edu (J.H.); alikens@unomaha.edu (A.L.); dkingston@unomaha.edu (D.K.)

**Keywords:** aquatic treadmill walking, underwater gait training, cerebral palsy rehabilitation, pediatrics

## Abstract

**Highlights:**

**What are the main findings?**

- This study showed improved sample entropy measures of the hip, knee, and ankle joints for the children with CP at slower than faster treadmill speeds in the aquatic treadmill compared to a dry treadmill environment.
- Sample entropy of the typically developing group’s knee and ankle joints improved in the aquatic treadmill environment compared to the dry treadmill.

**What is the implication of the main finding?**

- The findings imply that the aquatic treadmill environment could improve the walking regularity of children with cerebral palsy.

**Abstract:**

This block-randomized crossover study investigated how a speed-modulated aquatic treadmill (AT) impacts the walking biomechanics of pediatric gait. Eight cerebral palsy (CP) and fifteen typically developing (TD) children walked at normal, slow, and fast treadmill speeds in AT and dry treadmill (DT) conditions. The joint angles of participants were calculated from inertial measurement units to derive sample entropy (SE) measures that quantified the regularity or complexity of motion. A hierarchical statistical model revealed that the CP group had lower SE values for the hip, knee, and ankle joints in the AT and at slower than faster treadmill speeds. Only the SE values of the knee and ankle joints were impacted for the TD group. The lower SE values suggest improved regularity for participants at slower speeds and in the AT environment. This study highlights the potential of AT to improve the walking biomechanics of children with CP in acute exposure, but further work is needed to investigate the AT condition as a gait rehabilitation environment.

## 1. Introduction

Cerebral palsy (CP) causes complex motor disorders that may result from neurological and musculoskeletal deficits like muscle weakness, joint contractures, muscle spasticity, bony deformities, and an underlying loss of selective motor control [1,2]. Children with CP have different walking characteristics than typically developing (TD) children, as identified through 3D clinical gait analysis [1,3]. Some of the observed gait patterns have common clinical descriptions. For example, crouch gait has increased hip and knee flexion and ankle dorsiflexion during stance [4,5]. In contrast, equinus gait has pronounced plantarflexion during stance due to dominant spasticity of the gastrocnemius muscles [5,6]. The described walking patterns can vary widely and be complex in their presentations, sometimes leading to unstable walking in affected persons. Non-linear analytic measures like sample entropy have been used to characterize the biomechanics of their walking. Sample entropy assesses the regularity and predictability of time series data and can be used to quantify the degree of gait complexity [7,8]. It allows for the characterization of irregular walking patterns so that higher sample entropy values indicate greater complexity and less predictability in movement patterns, and lower values suggest more regular, predictable patterns that may reflect constrained or stereotypical movement. This measure can aid in understanding the neuromuscular control of locomotion concerning the regularity of motion for clinical populations in response to rehabilitation paradigms [7].

Gait patterns are highly variable across clinical classifications of CP [1,2], using the gross motor function classification system (GMFCS) I–V scale [9,10], but result in a loss of independent ambulation due to decreased overall mobility, specifically the higher spectrum of the GMFCS (III–V) [1,11]. Many rehabilitation interventions for CP focus on improving children’s walking dynamics to develop an efficient walking pattern and increase endurance [12,13]. Therapies such as treadmill walking that reinforce task-specific locomotor training have been encouraged and used for walking rehabilitation [14,15,16]. The aquatic treadmill (AT) is one variant used in rehabilitating children with CP, as evidence shows that treadmill walking, combined with the intrinsic characteristics of water, has the potential to improve the walking function of children with CP [17]. Buoyancy from partial submersion decreases the influence of gravity, and hydrodynamic drag resists segmental movement in water [18,19]. Combined, these characteristics of the aquatic environment provide a modifiable training dose that may better accommodate the different functional abilities of children with CP [19].

Growing evidence shows that water hydrodynamics can affect the gait characteristics of individuals walking on an AT [20]. Exercises performed in the aquatic environment improve motor function scores and are beneficial for improving balance control and walking performance of children with CP [21,22]. In some cases, hydrotherapy exercise interventions are stated as more advantageous than dry or land-based contemporaries [21,23] and can result in higher quality-of-life scores when used for rehabilitating children with CP [24]. Thus, we anticipate that integrating the benefits of an aquatic environment with treadmill walking could enhance rehabilitation outcomes for children with CP. However, research on the walking mechanics of children with CP when using AT is still limited. We lack a comprehensive understanding of fully utilizing partial body weight offloading from buoyancy, affected by water depth [18], and velocity-dependent hydrodynamic drag [25]. While evidence recommends waist-to-chest water depth for rehabilitation purposes [26], no known study has investigated how changes in treadmill walking speed could impact rehabilitation outcomes for children with CP in the aquatic environment. Filling this knowledge gap on walking speed could inform the therapeutic application and prescription of the AT for rehabilitating children with CP.

This study investigated changes in the walking dynamics of TD and children with CP when they walked at different treadmill speeds in AT and dry treadmill (DT) environments. Sample entropy quantified regularity from the lower limbs during three speed conditions in AT and DT. Understanding that a lower sample entropy value implies greater regularity or more predictable walking [7] we hypothesized that reducing the participants’ treadmill speed would initiate more regular walking dynamics in the AT compared to the DT environment. This hypothesis was motivated by expectations that water’s hydrodynamic drag would be better accommodated at reduced speeds than faster speeds when there would be increased mechanical turbulence. This should result in lower sample entropy measures for the participants in AT than DT conditions.

## 2. Materials and Methods

### 2.1. Participants

This observational study was a block-randomized crossover design study in which 8 children with CP (4:4 M: F, age = 12.4 ± 3.6 years (9–16 age range), height = 1.31 ± 13.96 m, weight = 45.56 ± 21.47 kg, BMI = 20.09 ± 6.51 kg/m^2^) and 15 TD children (7:8 M:F, age = 11.3 ± 4.1 years (6–18 age range), height = 1.46 ± 0.18 m, weight = 44.2 ± 16.8 kg, BMI = 19.91 ± 3.81 kg/m^2^) were recruited. The participants were recruited from an ongoing study. The CP group inclusion criteria considered GMFCS I-III, but we were only able to recruit GMFCS levels of I–II (6:2 ratio). The anatomical classification of their motor deficits was not considered before recruitment [3]. Other exclusion criteria were outside of enrollment age (6–18 years), those who received Botulinum Toxin Type A injections within the past four months, and those who cannot ambulate independently with or without using walking aids. Potential TD children who reported no prior pain or injuries to their lower extremities that required hospitalization within the past 12 months were included in the study. The institutional review board of the University of Nebraska Medical Center approved the study protocol, and participants and their guardians signed approved consent forms before participating in the study.

### 2.2. Data Collection

The protocol has been reported in a previous study [27]. Briefly, all participants were asked to walk at three randomized walking speed presentations for 3 min each in a DT before an AT environment. All participants walked at their normal comfortable speed (100%), slow speed (75% of normal speed), and fast speed (125% of normal speed), with the speeds determined distinctly for each of the AT and DT environments. Kinematic data were collected using waterproof Inertia Measurement Unit (IMU) sensors (WaveTrack Waterproof IMU, Cometa, Milan, IT; full-scale acc sensitivity = ±8 g; full-scale gyroscope sensitivity = 1000 dps; dimensions: 36 × 25 × 10 mm). Acceleration peaks from IMU sensors located on the feet were used to determine gait cycles of the joint angles during each walking trial. Heel strikes were determined using the greatest vertical acceleration peak, where initial contact occurred, and used to define gait cycles as initial contact to initial contact of the same foot [27]. The participants’ hip, knee, and ankle joint angles were obtained from the processing software for the IMU sensors, from which they were exported to MATLAB (2024b, The MathWorks, Natick, MA, US).

### 2.3. Data Processing

Data was exported from the “EMGandMotionTools” software (Version 8.11.0.0, Cometa S.r.l., Milan, Italy) and processed in MATLAB (2021a, The MathWorks, Natick, MA, US). Data were collected at a sampling frequency of 2000 Hz and downsampled to 142 Hz before sample entropy measures were derived in MATLAB (repository link; https://github.com/Nonlinear-Analysis-Core/NONANLibrary (accessed on 14 May 2025)). Sample entropy quantified the regularity of the sagittal plane angular positions of the hip, knee, and ankle joints of the limbs for all participants. To compute sample entropy, the data were time-normalized to 101 data points per gait cycle using linear interpolation. The first 60 Gait cycles were analyzed for each participant, with the vector length “*m*” set to 60% of the gait cycle and the tolerance set to 0.2 of the standard deviation of the time series data. The rationale to set *m* to 60 is that typical values for *m* (e.g., 2, 3) may not be meaningful for highly-sampled, smooth, continuous data like joint angles [28]. The 60% value was chosen so that vector matches within the sample entropy algorithm reflect a meaningful proportion of the data (e.g., the stance phase of gait).

### 2.4. Data Analysis

Statistical tests were performed in R (version 4.3.1 (2023-06-16 ucrt))/RStudio 2024.04.2 + 764 (R Core Team, 2021; RStudio Inc., Boston, MA, USA). A multilevel model [29] used an α = 0.05, and the “lmerTest” [30] (R package version 3.1-3) and “emmeans” [31] (R package version 1.8.8) packages were used in R/R-studio (Rstudio 2024.04.2 + 764). A sequential model-building approach following Raudenbush and Bryk’s [32] hierarchical modeling framework was adopted to investigate how three factors, walking environment (AT and DT), walking speed (Fast, Normal, and Slow), and group (CP and TD populations), impacted the sample entropy measures. For the joint angles, we built a series of linear mixed effects models with increasing complexity; the first model included only random participant effects, the second model tested the fixed effects for group, the third model tested the fixed effect of environment, the fourth model tested the fixed effect of speed, the fifth model tested the interaction of group and environment factors, the sixth model tested the interaction of group and speed factors, the seventh model tested the interaction of environment and speed interaction, and the final model tested the interaction of the three factors. This approach was used for the sample entropy measures for the ankles, knees, and hips. For each model comparison, we use likelihood ratio tests to evaluate improvement in model fit. The most parsimonious model with the lowest Akaike information criterion (AIC) value (with a difference of at least 2 points from simpler models) was selected as the best model. We then refit this model with restricted maximum likelihood estimation for final inference and conducted appropriate simple slope and post hoc tests based on the highest-order significant interaction. In likelihood ratio tests, the Type 1 error was set to 0.05.

## 3. Results

### 3.1. The Impact of Treadmill Environment, Speed, and Group (Population) on Sagittal Plane Hip Angles Sample Entropy Measures

**Figure 1:**
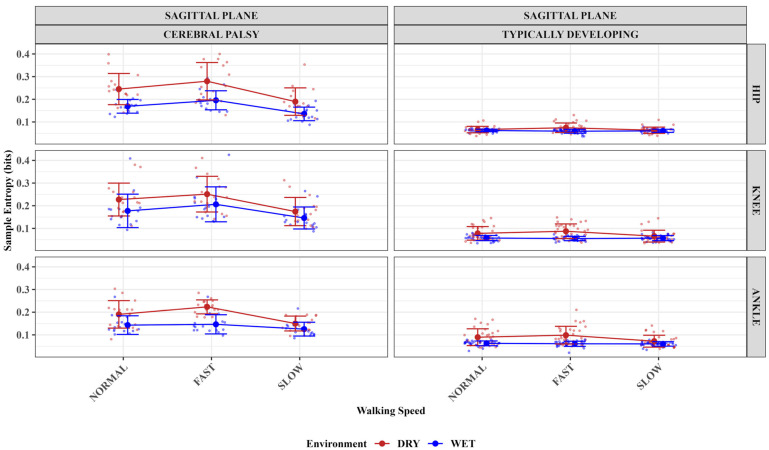
The impact of walking speed, treadmill environment, and group on the sample entropy values of the hip, knee, and ankle joints. The means are plotted as center dots, with each participant’s data points as small dots, and standard deviations appearing as error bars.

The best fitting model for sagittal plane hip angle sample entropy contained significant main effects of group (F (1, 23) = 258.12, *p* < 0.001), environment (F (1, 253) = 117.54, *p* < 0.001), and speed (F (2, 253) = 31.71, *p* < 0.001), with significant two-way interactions between group and environment pair (F (1, 253) = 77.85, *p* < 0.001) as well as group and speed pair (F (2, 253) = 31.71, *p* < 0.001). The best fitting model for hip motion in the sagittal plane included all main effects and all two-way interactions, but not the three-way interaction. Simple effects tests showed that only children with CP had significantly higher sample entropy in the DT environment compared to the AT environment (CP: F (1, 259.15) = 144.72, *p* < 0.001). For TD children, there was no significant difference between environments (TD: F (1, 259.15) = 2.86, *p* = 0.092). For children with CP, mean sample entropy was 0.2382 (DT) vs. 0.1667 (AT); for TD children, 0.0681 (DT) vs. 0.0607 (AT) (Figure 1). Similarly, speed effects were significant only for children with CP (F (2, 259.15) = 53.67, *p* < 0.001), but not for TD children (F (2, 259.15) = 0.36, *p* = 0.696). In the CP group, sample entropy increased with speed: slow (0.1626), normal (0.2071), and fast (0.2376). Post hoc comparisons showed significant differences between all speeds for CP (normal vs. fast: *p* < 0.001; normal vs. slow: *p* < 0.001; fast vs. slow: *p* < 0.001). In contrast, TD children maintained consistent levels of complexity across speeds (slow: 0.0621, normal: 0.0644, fast: 0.0667), with no significant differences between speed conditions (Figure 1).

### 3.2. The Impact of Treadmill Environment, Speed, and Group (Population) on Sagittal Plane Knee Angles Sample Entropy Measures

The sagittal plane knee angle model included the main effects of group (F (1, 23) = 200.88, *p* < 0.001), environment (F (1, 253) = 37.63, *p* < 0.001), and speed (F (2, 253) = 20.54, *p* < 0.001) factors. The model also had significant two-way interactions between group and environment (F (1, 253) = 4.16, *p* = 0.043) as well as between group and speed (F (2, 253) = 11.55, *p* < 0.001) factors, while the levels testing environment–speed interaction (F (2, 253) = 1.71, *p* = 0.18) and three-way interaction (*p* > 0.05) were not significant. The best-fit model tested the interaction of group–speed factors and had the lowest AIC (−944.75) value. Simple effects tests showed that both children with CP and typically developing (TD) children had significantly higher sample entropy in the DT compared to the AT environment (CP: F (1, 261.26) = 24.79, *p* < 0.001; TD: F (1, 261.26) = 11.68, *p* < 0.001). For the group–speed interaction, children with CP showed significant differences across speed conditions: fast walking produced higher entropy than normal speed (t (261.26) = −2.61, *p* = 0.026), normal speed produced higher entropy than slow walking (t (261.26) = 4.17, *p* < 0.001), and fast walking produced higher entropy than slow walking (t (261.26) = 6.77, *p* < 0.001).

### 3.3. The Impact of Treadmill Environment, Speed, and Group (Population) on Sagittal Plane Ankle Angles Sample Entropy Measures

The best-fitting model for the sagittal plane ankle motion sample entropy had the lowest AIC (−1167.07) value and included main effects of group (F (1, 23) = 117.71, *p* < 0.001), environment (F (1, 253) = 133.91, *p* < 0.001), and speed (F (2, 253) = 29.88, *p* < 0.001). This model included significant two-way interactions between group and environment (F (1, 253) = 13.54, *p* < 0.001), group and speed (F (2, 253) = 8.99, *p* < 0.001), as well as environment and speed (F (2, 253) = 11.99, *p* < 0.001), while the level testing the three-way interaction was not significant (*p* > 0.05). Simple effects showed that both children with CP and TD children had significantly higher sample entropy in the DT compared to the AT environment (TD: F (1, 263.41) = 42.995, *p* < 0.001; CP: F (1, 263.41) = 85.652, *p* < 0.001). Speed effects were also significant for both groups (TD: F (2, 263.41) = 4.292, *p* = 0.015; CP: F (2, 263.41) = 26.329, *p* < 0.001). In the CP group, sample entropy increased with speed (slow (0.1377), normal (0.1667), and fast (0.1850 with post hoc comparisons showing significant differences between all speeds (normal vs. fast: *p* = 0.016; normal vs. slow: *p* < 0.001; fast vs. slow: *p* < 0.001). In the TD group, the only significant difference was between fast and slow speeds (*p* = 0.014). For the environment–speed interaction, the DT environment produced significantly higher sample entropy values than the AT environment at all speeds (normal: F = 41.796, fast: F = 99.636, slow: F = 10.218; all *p* < 0.002).

### 3.4. Summaries

**Figure 2:**
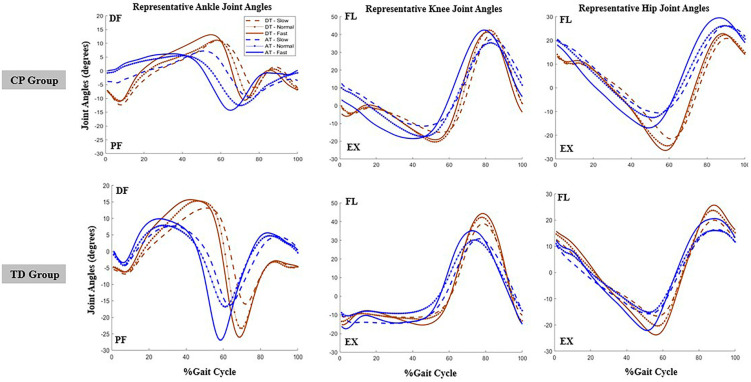
Image shows the time series joint angle plots for a representative CP (**1st row**) and TD (**2nd row**) child in the aquatic treadmill and dry treadmill environment, time normalized to stride: AT—aquatic treadmill, DT—dry treadmill, CP—Cerebral palsy, and TD—Typically developing, PF—plantarflexion, DF—dorsiflexion, FL—flexion, EX—extension.

Table 1 below summarizes the best-fit models for sample entropy analysis of hip, knee, and ankle joint angles. Table 2 shows the mean and standard deviation of the sample entropy values for all participants grouped by speed, group, and environmental factors. Figure 2 below shows representative plots of the joint angles for the hip, knee, and ankle for children with cerebral palsy and typically developing children.

## 4. Discussion

This study investigated how underwater treadmill walking at fast, normal, and slow speeds impacted the lower limb motion regularity of TD and children with CP compared to walking on a dry treadmill. Sample entropy measures, derived from the participants’ lower extremity joint angles, were used to assess how the treadmill conditions impacted the outcome measure. Our findings suggest lower sample entropy measures in the AT compared to DT treadmill environment and at slower compared to fast speeds. These findings were consistent for hip, knee, and ankle joints. Surprisingly, only the sample entropy of the hip motion of TD children was not impacted by treadmill walking speeds and the different walking environments. As hypothesized, the participants had lower sample entropy measures in the AT environment and at lower speeds, except for the TD participants’ Hip angle, which contradicts the hypothesis.

The results of this study corroborated existing evidence that shows aquatic environments have great rehabilitative potential for children with CP [17]. For the sagittal plane hip motion, the results showed that complexity in children with CP is highly sensitive to both walking environment and speed changes, with the most irregular or complex movements occurring during fast walking on a dry surface. In contrast, TD children maintained consistent levels of movement complexity regardless of environmental conditions or walking speed. This pattern demonstrates a fundamental difference in motor control adaptability between the two groups, with CP showing environment-dependent and speed-dependent modulation of movement complexity not observed in TD children. These findings regarding hip motion regularity were consistent with the participants’ knee and ankle motion regularity. The sagittal plane knee motion complexity in children with CP also showed sensitivity to both walking environment and speed, with the most complex movements occurring during fast walking on a dry surface, followed by normal speed, and lowest at slow speed.

The DT environment consistently produced higher entropy values than the AT environment across all speeds for both groups, though the effect was stronger in the CP group. Conversely, TD children maintained consistent levels of movement complexity regardless of walking speed across all conditions, a consistent finding with the hip motion complexity. Only the ankle motion complexity was different for TD children. Here, the TD children show a smaller increase in complexity at higher speeds, with a significant difference only between fast and slow speeds. This corroborates studies that show that sagittal motion ankle movement tends to be influenced by speed modulation [33]. Also, both the CP and TD groups experienced higher complexity in the DT environment than in the AT, with a larger effect in children with CP. Decreasing walking speed resulted in lower sample entropy, especially in the CP group, which shows clear decreases from fast to normal to slow speeds.

The decreased sample entropy observed in the AT environment for participants of this study infers improved movement regularity or lesser complexity, which may refer to improved walking dynamics. This may indicate that the children with CP walked with improved walking dynamics in the AT compared to the DT environments and at slower compared to faster speeds. The available TD data served as a basis for comparison, indicating that children with CP generally exhibited higher sample entropy than their TD peers. Notably, these sample entropy values improved in the AT compared to the DT environment. These results suggest the AT environment may improve walking in children with CP, and these results were no different for the TD group.

The gait of children with CP is impaired, such that they experience a more variable and asymmetrical gait pattern than their TD peers [34,35]. Children with CP may experience decreased muscle strength, selective muscle activation, and limited proprioception, which could translate into poor walking dynamics and balance problems [36,37]. Water’s buoyancy has been shown to decrease the gravitational effect, enabling the relaxation of muscles and aiding postural control, while the aquatic environment also increases sensory feedback [21,38]. These factors may have improved motor control for children with CP and stimulated improved walking dynamics [21], as observed in this current study. This can result in an overall improved gross motor function and walking ability [21]. Also, the hydrodynamic drag, influenced by segmental velocity, played a role in the participants’ movement in the AT environment, as evidence shows that the aquatic resistance or drag is increased up to four times at faster speeds [39].

Faster speeds also increase the mechanical turbulence of the AT environment and could be the reason for the improved walking dynamics at slower speeds compared to faster speeds. The participants may find it easier to navigate the AT environment in the less turbulent aquatic environment at slower speeds than faster treadmill speeds. This finding may influence rehabilitation decisions as treadmill walking speeds may be modulated for children with CP in the AT environment to modulate a rehabilitation environment that can improve their walking dynamics.

Findings from this study need to be interpreted considering its limitations. First, we did not consider the different gait phases while reporting the results of this study, as waterproof IMUs do not have a validated approach to segment the gait cycle into these phases. It is unclear how buoyancy would impair gait event detection using accelerometry-based calculations. Also, the low sample size of children with CP prevents the exploration of the different presentations of this heterogeneous condition [40], considering that the included participants were GMFCS I-II, and the anatomical classification of their motor deficit was not considered. Including a broader CP population would provide a more generalized understanding of how AT impacts these children over different classification levels. It could also aid in narrowing down how AT influences the gait of an impaired limb compared to a less impaired or unimpaired limb, especially in monoplegia or paraplegia presentations, as the aquatic environment can accommodate individuals with different functional capacities [41]. Future studies can address these limitations to ensure more comprehensive findings.

## 5. Conclusions

This study shows the potential of an AT walking environment when used for walking in children with CP compared to the DT counterpart. Aquatic treadmill walking influenced a more regular walking motion in children with CP. AT may be used to create an environment where CP children may walk with better walking dynamics when compared to walking on the DT. A longitudinal study where AT is used in a structured rehabilitation program is an essential next step to assess the retention of the observed impacts of the AT environment on children with CP.

## Figures and Tables

**Table 1:** Summary of selected levels for Statistical Multilevel model steps for joint angles sample entropy measures.

Joint-Direction	Best Model	Model Index	AIC	BIC	logLik	*p*
*Sagittal Hip*	*Model 6: +Environment:Speed*	7	−1116.71	−1073.27	570.36	**<0.001**
*Sagittal Knee*	*Model 5: +group:Speed*	6	−944.75	−908.55	482.38	**<0.001**
*Sagittal Ankle*	*Model 6: +Environment:Speed*	7	−1167.07	−1123.63	595.54	**<0.001**

Note: AIC—Akaike Information Criteria, BIC—Bayesian Information criteria, and logLik—Log-likelihood.

**Table 2:** Means and standard deviations of sample entropy of participants for the hip, knee, and ankle joints.

Joint	Group	Speed	Environment	Mean	Std_dev
Hip	CP	Fast	DT	0.28	0.08
Hip	CP	Fast	AT	0.2	0.04
Hip	TD	Fast	DT	0.07	0.02
Hip	TD	Fast	AT	0.06	0.01
Hip	CP	Normal	DT	0.25	0.07
Hip	CP	Normal	AT	0.17	0.03
Hip	TD	Normal	DT	0.07	0.01
Hip	TD	Normal	AT	0.06	0.01
Hip	CP	Slow	DT	0.19	0.06
Hip	CP	Slow	AT	0.14	0.03
Hip	TD	Slow	DT	0.06	0.01
Hip	TD	Slow	AT	0.06	0.01
Knee	CP	Fast	DT	0.25	0.08
Knee	CP	Fast	AT	0.21	0.08
Knee	TD	Fast	DT	0.09	0.03
Knee	TD	Fast	AT	0.06	0.01
Knee	CP	Normal	DT	0.23	0.07
Knee	CP	Normal	AT	0.18	0.07
Knee	TD	Normal	DT	0.08	0.03
Knee	TD	Normal	AT	0.06	0.01
Knee	CP	Slow	DT	0.17	0.06
Knee	CP	Slow	AT	0.15	0.05
Knee	TD	Slow	DT	0.07	0.03
Knee	TD	Slow	AT	0.06	0.01
Ankle	CP	Fast	DT	0.22	0.03
Ankle	CP	Fast	AT	0.15	0.04
Ankle	TD	Fast	DT	0.1	0.04
Ankle	TD	Fast	AT	0.06	0.01
Ankle	CP	Normal	DT	0.19	0.06
Ankle	CP	Normal	AT	0.14	0.04
Ankle	TD	Normal	DT	0.09	0.04
Ankle	TD	Normal	AT	0.06	0.01
Ankle	CP	Slow	DT	0.15	0.03
Ankle	CP	Slow	AT	0.13	0.03
Ankle	TD	Slow	DT	0.07	0.03
Ankle	TD	Slow	AT	0.06	0.01

Note: CP—cerebral palsy, TD—typically developing, Std-dev—standard deviation, DT—dry treadmill, and AT—aquatic treadmill.

## Data Availability

The datasets generated during and/or analyzed during the current study are available from the corresponding author upon reasonable request.
